# Production of Pectinolytic Enzymes by the Yeast *Wickerhanomyces anomalus* Isolated from Citrus Fruits Peels

**DOI:** 10.1155/2013/435154

**Published:** 2013-04-17

**Authors:** María A. Martos, Emilce R. Zubreski, Oscar A. Garro, Roque A. Hours

**Affiliations:** ^1^Facultad de Ciencias Exactas, Químicas y Naturales, Universidad Nacional de Misiones, Felix de Azara 1552, N3300LQH Posadas, Argentina; ^2^Universidad Nacional del Chaco Austral, Comandante Fernández 755, H3700LGO Presidencia Roque Sáenz Peña, Argentina; ^3^Centro de Investigación y Desarrollo en Fermentaciones Industriales (CINDEFI, UNLP, CONICET La Plata), Facultad de Ciencias Exactas, Universidad Nacional de la Plata, Calle 47 y 115, B1900ASH La Plata, Argentina

## Abstract

*Wickerhamomyces anomalus* is pectinolytic yeast isolated from citrus fruits peels in the province of Misiones, Argentine. In the present work, enzymes produced by this yeast strain were characterized, and polygalacturonase physicochemical properties were determined in order to evaluate the application of the supernatant in the maceration of potato tissues. *W. anomalus* was able to produce PG in liquid medium containing glucose and citrus pectin, whose mode of action was mainly of endo type. The supernatant did not exhibit esterase or lyase activity. No others enzymes, capable of hydrolyzing cell wall polymers, such as cellulases and xylanases, were detected. PG showed maximal activity at pH 4.5 and at temperature range between 40°C and 50°C. It was stable in the pH range from 3.0 to 6.0 and up to 50°C at optimum pH. The enzymatic extract macerated potato tissues efficiently. Volume of single cells increased with the agitation speed. The results observed make the enzymatic extract produced by *W. anomalus* appropriate for future application in food industry, mainly for the production of fruit nectars or mashed of vegetables such as potato or cassava, of regional interest in the province of Misiones, Argentine.

## 1. Introduction

Enzymes hydrolyzing pectic substances, which contribute to the firmness and structure of plant cells, are known as pectinolytic enzymes or pectinases. Based on their mode of action, these enzymes include polygalacturonase (PG), pectinesterase (PE), and lyases (pectinlyase (PL) and pectatelyase (PAL)). PG, PL, and PAL are depolymerizing enzymes, which split the *α*-(1,4)-glycosidic bonds between galacturonic monomers in pectic substances either by hydrolysis (PG) or by *β*-elimination (PL, PAL). PG catalyzes the hydrolytic cleavage of the polygalacturonic acid chain while PL performs a transeliminative split of pectin molecule, producing an unsaturated product. PE catalyzes the de-esterification of the methoxyl group of pectin, forming pectic acid [1, 2]. There are two types of PGases with different technological applications: exopolygalacturonases (exo-PG) that break down the distal groups of the pectin molecule, reducing chain length relatively slowly, and endopolygalacturonases (endo-PG) which act randomly on all the links in the chain, reducing molecular dimensions and viscosity more rapidly [3].

Pectinolytic enzymes play an important role in food technology, mainly in the processing of fruit juices and wines and in the maceration of plant tissue. Maceration is a process by which organized tissue is transformed into a suspension of intact cells, resulting in pulpy products used in the food industry for the production of fruit nectars as pears, peaches, apricots, strawberries, and vegetables mashed such as potatoes, carrots, red pepper, and others that are used in babies and seniors foods [4]. For such purposes, only the intercellular cementing material that holds together cells and some portion of primary plant cell walls should be removed without damage to adjacent secondary cell walls, to help avoid cell lysis, keeping nutritional properties of food [5]. For this reason, cellulases in the enzyme mixture are undesirable [6]. 

The stability of pectinases is affected by both physical parameters (pH and temperature) and chemical parameters (inhibitors or activators). Enhancing the stability and maintaining the desired level of activity over a long period are two important points considered for an efficient application of these enzymes [7].

Pectinases used in the food industry are commercially produced by *Aspergillus niger*. Commercial preparations of fungal origin contain a complex mixture of different enzymes with pectinolytic activity, including PGases, lyases, the undesirable PE, and others enzymes. Yeasts have advantages compared to filamentous fungi, because they are unicellular, the growth is relatively simple, and usually yeasts do no secret PE [8].

A yeast isolated from citrus fruit peels in the province of Misiones (Argentine) and identified as *Wickerhamomyces anomalus*, recent reclassification of the species *Pichia anomala* [9], produced pectinolytic enzymes in liquid medium containing glucose and citrus pectin as carbon and energy sources and inductor, respectively. In the present work, enzymes produced by this wild yeast strain were characterized, and physicochemical properties of polygalacturonase were determined by the study of the effect of temperature and pH on its activity and stability, in order to evaluate the application of the supernatant in the maceration of potato tissues. 

## 2. Materials and Methods

### 2.1. Microorganism

*W. anomalus* was isolated from citrus fruit peels in the province of Misiones (Argentine). 

### 2.2. Culture Media

*YM Medium*. Yeast extract (Sigma), 5 g/L; tryptone (Difco-Becton Dickinson & Co.), 5 g/L; glucose (Britania), 10 g/L; agar (Britania), 15 g/L, pH 5.0.

*YNB Medium*. Yeast Nitrogen Base (YNB, Difco-Becton Dickinson & Co.), 6.7 g/L; glucose (Britania), 5 g/L; citrus pectin (Parafarm), 5 g/L; pH 5.0.

Citrus pectin was washed with a 70% (v/v) ethanol-HCl (0.05 N) solution to remove soluble sugars [10].

All components of media were autoclaved (121°C, 15 min) except in the case of YNB solution which was sterilized separately by filtration through a cellulosic filter paper (0.22 *μ*m, Sartorius). 

### 2.3. Production of Pectinolytic Enzymes in Submerged Fermentation

Five hundred millilitre Erlenmeyers flasks with 95 mL of YNB medium were inoculated with 5 mL of an appropriate dilution of a suspension of the microorganism (DO_620_ = 0.96), grown in *YM medium* (30°C, 24 h). The Erlenmeyers flasks were incubated at 30°C for 10 h on a rotary shaker at 180 rpm. The biomass was separated by centrifugation at 4000 rpm, for 10 min at 5°C. The culture medium supernatant was frozen at –18°C and used as source of extracellular enzymes (named enzymatic extract, EE). The assay batch cultures were run in triplicate and mean values were calculated. 

### 2.4. Enzyme Assays

*Polygalacturonase (PG).* PG activity was assayed by measuring the reducing groups released by dinitrosalicylic acid method [11]. A calibration curve was made using galacturonic acid (GA, Sigma) as standard. One unit of PG was defined as the amount of enzyme which releases 1 *μ*mol of GA per minute.

*Xylanase and Cellulose.* Xylanase and cellulase activities were assayed as was PG activity except for the use of xylan (Sigma) and carboxymethylcellulose (Sigma), respectively, as substrates. Xylose (Sigma) and glucose (Sigma) were used as standard for xylanase and cellulase, respectively.

*Pectinlyase (PL).* PL was assayed by monitoring the increase in absorbance at 235 nm of citrus pectin (Sigma) solution, as described by [12]. One unit of PL activity was defined as the amount of enzyme which produces an increase of one unit of absorbance in the conditions of the assay.

*Pectatelyase (PAL).* PAL was assayed as was PL activity except for the use of PGA (Sigma) as the substrate.

*Pectinesterase (PE).* PE activity was determined by color change of a pH indicator (bromocresol green) added to the reaction mixture, due to carboxyl groups being released during the reaction. As a substrate, it was used 0.5% (w/v) citrus pectin (Sigma) in water, pH 5.0 [13]. 

### 2.5. Mode of Action of PG

The endo- or exo-mode of action of PG was determined by measuring the formation of reducing groups and the changes in viscosity of 5 g/l PGA (Sigma) solution in AcB (0.2 M, pH 5.0), at 37°C. 

For thin-layer chromatography (TLC) analysis of PGA degradation products, heat inactivated samples were spotted (10 *μ*L) on aluminium sheets (silica gel 60 F254, Merck) and the chromatography performed by using the ascending method with n-butanol : acetic acid : water (9 : 4 : 7, v/v/v) as the solvent system. Detection was accomplished by spraying the dried plate with 3% (w/v) phosphomolybdic acid dissolved in 10% (v/v) sulfuric acid in ethanol followed by heating at 105°C for 5 min. GA was used as standard [14].

An endo-PG is characterized by a strong reduction in viscosity (e.g., 50%) with a concomitantly low release of reducing groups and the first products are oligomers, whereas an exo-PG has to hydrolyse greater than 20% of the glycosidic linkages to obtain an equivalent viscosity reduction and the first degradation products are monomers or dimmers [15–17].

### 2.6. Effect of pH on Polygalacturonase Activity and Stability

The effect of pH on PG activity was determined by incubating the reaction mixture at pH values ranging from 3.5 to 6.0, under standard enzyme assay conditions. 

The pH stability of the enzyme was evaluated by measuring the residual activity, under standard enzyme assay conditions, after incubating the EE without substrate for 24 h at 4°C at various pH from 2.0 to 8.0. The buffers employed in these measurements were citrate/phosphate buffer, for pH 2.0–3.5 and 6.0–8.0 and AcB (0.2 M) for pH 4.0–5.5. All the experiments were conducted in triplicate and the results show the mean values of the activities.

### 2.7. Effect of Temperature on Polygalacturonase Activity and Stability

The effect of reaction temperature on PG activity was tested by incubating the reaction mixture at temperatures from 15°C to 60°C, at pH optimum, under standard enzyme assay conditions. 

The thermostability of the enzyme was determined by measuring the residual activity, under standard enzyme assay conditions, after incubating the EE without substrate at temperatures from 45°C to 60°C at pH optimum.

All the experiments were conducted in triplicate and the results show the mean values of the activities.

### 2.8. Assay of Maceration Activity

The effect of reaction time and shaking on potato maceration and final yield of single cells were evaluated. 

Potatoes, purchased from a local market, were used for tests of maceration with the EE of *W. anomalus*. Potatoes were peeled and cut into pieces measuring 3-4 mm on each side. Enzymatic maceration was carried out in 125 mL Erlenmeyer flasks containing 5 mL of AcB (0.2 M) at pH optimum, 3 g of vegetable tissue, and 5 mL of EE. Flasks were incubated, at optimum temperature, up to 300 min in a shaker at different agitation speed. The whole content of the flasks was filtered through a 20 mesh screen into a 10 mL graduated conical test tube. Suspension of single cells was kept at 5°C for 4 h. The volume of single cells decanted was measured. Residual undegraded plant tissue was dried at 80°C until constant weight and then weighed. As a control, blanks were prepared with heat-denatured enzymes. Microscopic observations of the maceration process were also done [6]. Each experience was run in triplicate and mean values were calculated.

## 3. Results and Discussion

### 3.1. Extracellular Enzyme Activities

*W. anomalus* was able to produce PG (~51 U/mL) in liquid medium containing YNB, glucose, and citrus pectin. The supernatant (10x) did not exhibit esterase or lyase activity. No other enzymes, capable of hydrolyzing cell wall polymers, such as cellulases and xylanases, were detected. 

These results are in agreement with the observation of several authors who reported that the most common enzyme found to be secreted by pectinolytic yeasts is PG [17, 18]. Schwan et al. [16] reported that four yeast strains isolated from cocoa fermentations (*Kluyveromyces marxianus*, *K. thermotolerans,* and *Saccharomyces cerevisiae* var. *chevalieri*) showed extracellular PG activity, and neither PE nor lyases were detected in culture filtrates. Eight wine yeast strains of *Saccharomyces* sp. produced PG but none of them produced PL or PAL [19]. Masoud and Jespersen [20] reported that yeasts predominant during coffee processing (six strains of *Pichia anomala*, four strains of *P. kluyveri,* and two strains of *Hanseniaspora uvarum*) were found to secrete PG but no PL or PE was found to be produced by the yeasts examined.

### 3.2. Mechanism of Action of PG

**Figure 1:**
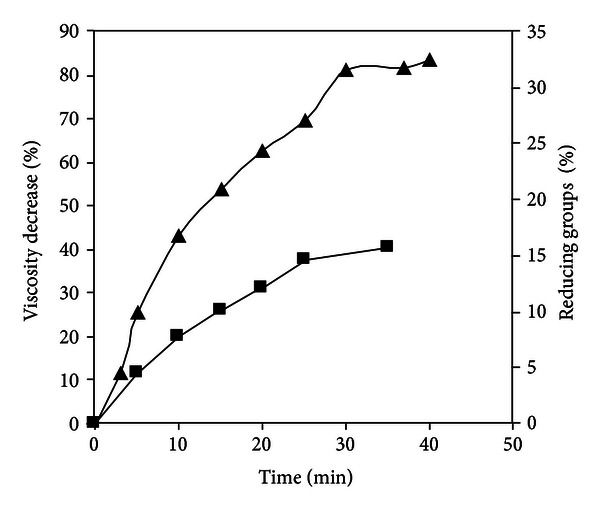
Degradation of polygalacturonic acid with the extract enzymatic of *W. anomalus*. Symbols: (-▲-) viscosity decrease, (-■-) reducing groups.

**Figure 2:**
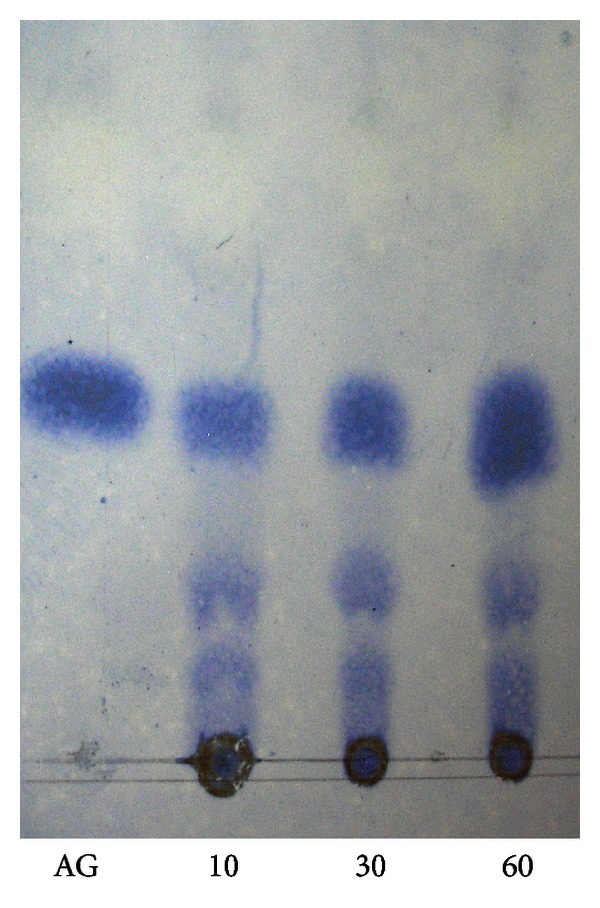
Thin-layer chromatography of the degradation products during enzymatic digestion of PGA solution with the extract enzymatic of *W. anomalus*. Numbers below each line indicate the reaction time.

Figure 1 shows the decrease in viscosity and increase in reducing groups as a function of time of a PGA solution by the EE of *W. anomalus* and Figure 2, shows the thin-layer chromatography of the degradation products during enzymatic digestion.

Figure 1 shows that the viscosity of the substrate decreased 50% when only 9% of the glycosidic bonds were split. The TLC analysis of the products of PGA hydrolysis indicates that mono-, di-, and tri-galacturonanos and higher oligosaccharides were produced from the initial stages of the hydrolysis and accumulated throughout the incubation period. PG did not seem able to attack dimers and trimers as these products were accumulated throughout the incubation period (Figure 2). From these results, it can be deduced that PG of *W. anomalus* acts by an endo-splitting mechanism, so it is an endo-PG (EC 3.2.1.15) [17, 21]. 

Pectinolytic enzymes from yeasts are mainly endo-PG [22]. This observation is in agreement with those reported for *K. marxianus* [16], *S. cerevisiae* 1389 and *S. cerevisiae* IMI-89 [23], *K. wickerhamii* [24] and *Thermoascus aurantiacus* CBMAI-756 [25] which acted by an endo mechanism.

### 3.3. Effect of pH on PG Activity and Stability

**Figure 3:**
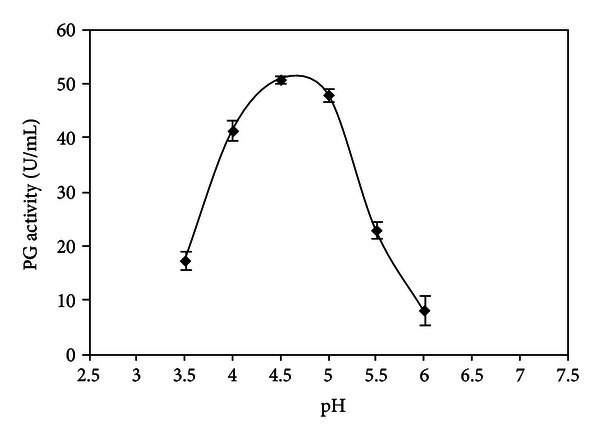
Effect of pH on PG activity produced by *W. anomalus*.

**Figure 4:**
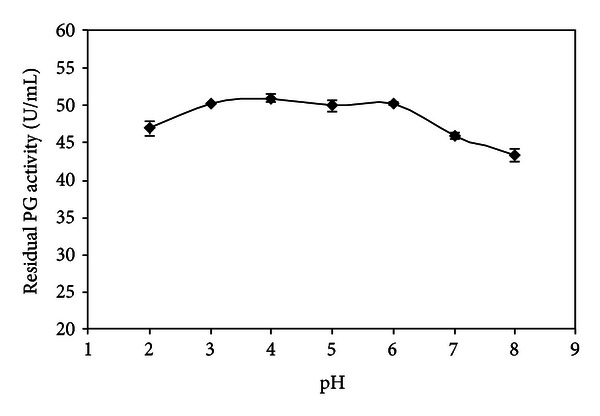
Effect of pH on PG stability produced by *W. anomalus*.

The effect of pH on PG activity and stability produced by *W. anomalus* is shown in Figures 3 and 4, respectively. 

PG secreted by *W. anomalus* exhibited maximal activity at pH 4.5. At pH 4.0 and 5.0, PG activity values were 81% and 94%, respectively (Figure 3). 

Figure 4 shows that the enzyme was stable at a pH range from 3.0 to 6.0, after incubation time of 24 h at 5°C. Analysis of variance (*P* < 0.05) revealed no significant differences between these values. The enzyme retained 92% and 85% of its activity at pH 2.0 and 8.0, respectively. 

Blanco et al. [18] reported that yeasts PGases exhibit an optimum pH in the acidic region between 3.5 and 5.5. This is in accordance with that reported for PGases produced by *S. cerevisiae* IM1-8b; *S. cerevisiae* 1389 [26]; *K. wickerhamii* [24]; *S. cerevisiae* UCLMS-39, yeast isolated from wine ecosystems [3]; *K. wickerhamii* strain 185 and *K. marxianus* strain 166, both yeasts from tropical fruits [8], and PGases from *K. marxianus* CCT 3172, *P. anomala* S16, and *P. kluyveri* S13Y4, yeasts predominant during coffee processing [20].

### 3.4. Effect of Temperature on PG Stability

**Figure 5:**
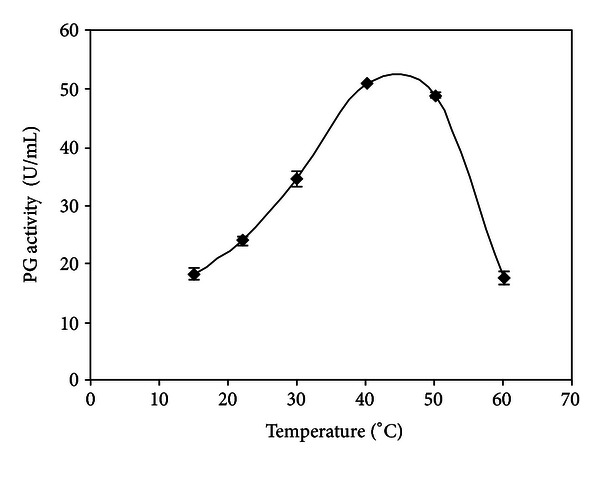
Effect of temperature on PG activity produced by *W. anomalus*.

**Figure 6:**
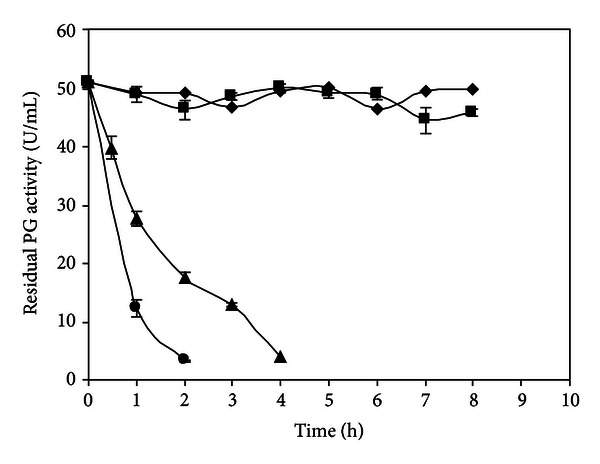
Effect of temperature on PG stability produced by *W. anomalus*. Symbols: (◆) 45°C, (■) 50°C, (▲) 55°C, (●) 60°C.

The effect of temperature on PG activity and stability produced by *W. anomalus* is shown in Figures 5 and 6, respectively.

Figure 5 shows that PG activity was higher in a temperature range between 40°C and 50°C at pH 4.5. Analysis of variance revealed no significant differences between these values (*P* < 0.05). The value of PG activity at 40°C was 1.5 times higher than the value obtained at 30°C. 

PGases isolated from different microbial sources differ markedly from each other with respect to their physicochemical properties; most have optimal temperature range of 30°C–50°C [1]. PG produced by *K. marxianus* [16], *K. marxianus* CCT 3172, and *P. anomala* S16 [20] exhibited maximum activity at 40°C and that of *K. Wickerhamii* [24] and *P. kluyveri* S13Y4 [20] at 50°C.

Figure 6 shows that in the absence of substrate, PG was stable at 45°C and 50°C during 8 h of incubation, at optimum pH. At 55°C, the enzymatic activity decreased and retained 78% and 54% of the initial activity after 30 min and 1 h of incubation, respectively. At 60°C thermal inactivation rate was higher and after 1 h of incubation the residual activity was only 24%.

The termoestabilidad of PG produced by *W. anomalus* was similar to that reported for PGases from other yeasts like *S. cerevisiae* IM1-8b and *S. cerevisiae* 1389 which were quite stable in the 20–50°C temperature range but were inactivated (80%) within 5 min at 55°C [23]. 

The knowledge of enzyme deactivation and stability is important to maintain the desired level of enzyme activity over a long period of time and improve its stability for an efficient application in an industrial process [25]. Besides after any application, the enzyme has to be inactivated, so the knowledge of thermal inactivation has great importance [27].

### 3.5. Assay of Maceration Activity

**Figure 7:**
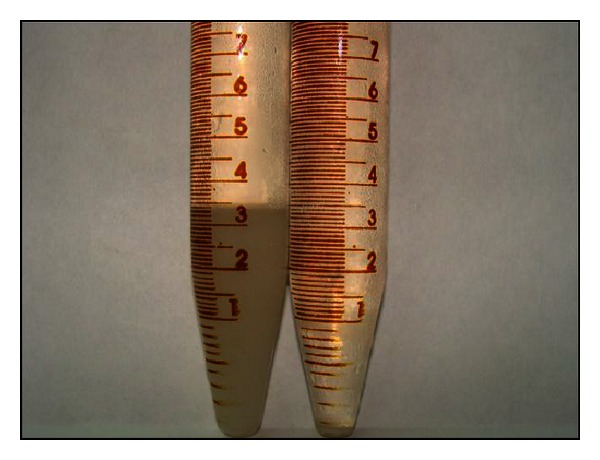
Decanted free cells after enzymatic maceration of potato tissue with the EE of *W. anomalus*. Left: negative control.

**Figure 8:**
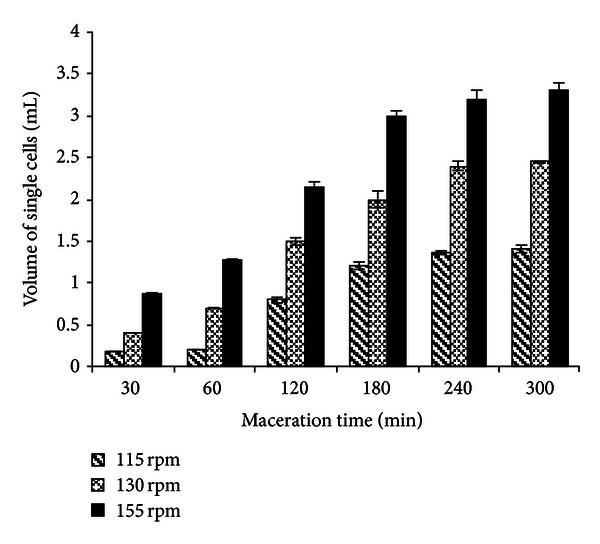
Volume of single cells as a function of reaction time at different agitation speeds, during maceration of potato tissues with enzymatic extract of *W. anomalus*.

**Figure 9:**
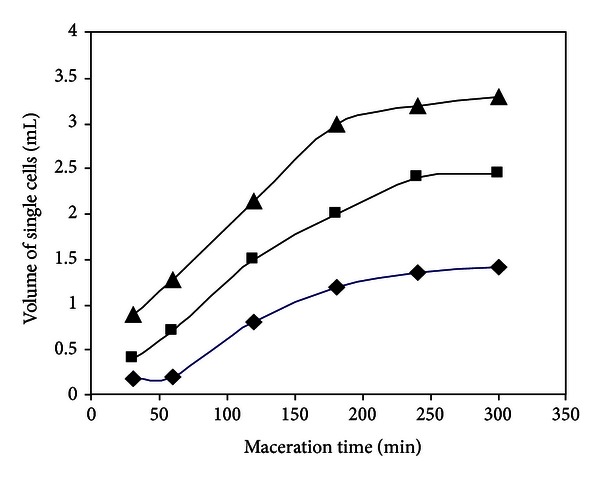
Kinetics of maceration process from potato tissues with the enzymatic extract of *W. anomalus*. Symbols (rpm): 115 (◆), 130 (■), 155 (▲).

Figure 7 shows a photograph of conical tubes containing the decanted material (released cells) after enzymatic maceration of potato tissue with EE of *W. anomalus* at 45°C (within the range of PG stability) and pH 4.5 (optimum pH of PG). Effect of shaking on single-cell production is shown in Figure 8 and kinetics of single-cell production is present in Figure 9.

Figure 7 shows that the EE macerated plant tissue efficiently, and maceration blanks yielded negligible values. Blanks with the inactivated enzyme showed that, in all cases, the effect was caused mainly by maceration activity of PG present in the EE and not by mechanical (shear) effects only.

Volume of single cells increased with reaction time at the three agitation speeds tested (115, 130, and 155 rpm) (Figure 8). 

The rate of maceration at 115 rpm was low, and it increased at higher agitation speed (Figure 9). The rate of maceration at 155 rpm was higher at all times tested and yielded large amounts of single cells. At this agitation speed, volume of single cells increased almost linearly up to 180 min, suggesting that longer reaction times may not be necessary to achieve maximum maceration yields. 

**Figure 10:**
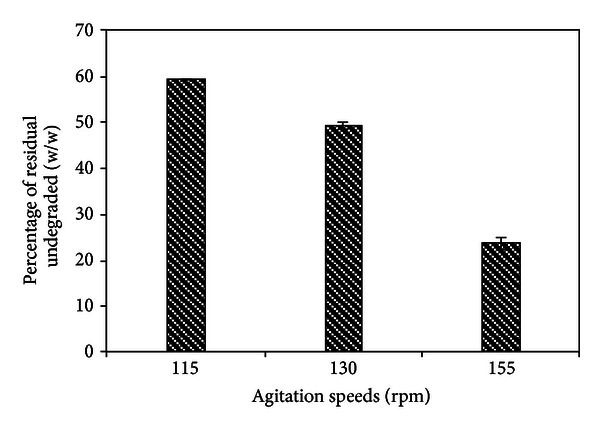
Residual undegraded of potato tissue as a function of agitation speeds after 300 min of time reaction with the enzymatic extract of *W. anomalus*.

The percentage of residual undegraded plant tissue produced at the end of the maceration process (300 min) at three agitation speeds is shown in Figure 10. 

Figure 10 shows that it was obtained a 59.6%, (w/w), 49% (w/w), and 24.9% (w/w) of undegraded residue at 115, 130, and 155 rpm, respectively. Therefore the efficiency of the enzyme in macerating potato was higher at 155 rpm (about 75% of plant material was converted into cell free) and the yield of single cells produced was high. It would be important because the main purpose of enzymatic maceration is to maximize conversion of plant tissues into single cells. Consequently, amounts of the plant material that resisted enzymatic reaction should be minimized to optimize yield [6]. In contrast, at 115 rpm most of the initial material remained as an insoluble residue and the yield single cell volume was low. 

The ability to release pectin from protopectin, leading to the maceration of plant tissues, depends on two factors: the chemical structure of the substrate and the ability of the enzyme to reach and degrade the specific site where the reaction takes place. Once partial depolymerization of the middle lamella had occurred, and a shear force was needed to transform the plant material into a suspension of loose cells [6].

*P. anómala* produces PG with maceration activity of potato tissues. Several PPases from bacterial, yeast or fungal origins have been isolated and characterized [28, 29].

## 4. Conclusions

The results showed that *W. anomalus* has a pectolytic system which consists essentially of an enzyme with polygalacturonase activity, whose mode of action is mainly of endo-type. Other enzymes such as PE, lyases, cellulases, and xylanases were not detected. PG exhibited an optimum pH in the acidic region and was stable up to 50°C, suited to most fruit and vegetable processing applications. The enzyme was responsible of the maceration of potato tissue observed. 

This yeast is able to produce only an enzyme with polygalacturonase activity, making the downstream processing easier if pure enzyme is needed since separation from other enzymes is not required. Therefore polygalacturonase like the one characterized in this study could be a potential candidate for different applications in food industry, mainly in the softening of vegetables for the preparation of babies and seniors foods or for the production of dehydrated mashed cassava of regional interest in the province of Misiones, Argentine.
